# Writing In and Reading ICU Diaries: Qualitative Study of Families' Experience in the ICU

**DOI:** 10.1371/journal.pone.0110146

**Published:** 2014-10-16

**Authors:** Maité Garrouste-Orgeas, Antoine Périer, Philippe Mouricou, Charles Grégoire, Cédric Bruel, Sandie Brochon, François Philippart, Adeline Max, Benoit Misset

**Affiliations:** 1 Medical-Surgical ICU, Saint Joseph Hospital Network, Paris, France; 2 IAME, UMR 1137, Paris Diderot University, Sorbonne Paris Cité, Paris, France; 3 Maison des Adolescents, University Hospital Cochin, Paris, France; 4 INSERM U-669, University Paris Sud and University Paris Descartes, UMR-S0669, Paris, France; 5 Management department, ESSCA School of Management PRES UNAM, Boulogne- Billancourt, France; 6 University Paris Descartes, Paris, France; University of Stirling, United Kingdom

## Abstract

**Purpose:**

Keeping an ICU patient diary has been reported to benefit the patient's recovery. Here, we investigated the families' experience with reading and writing in patient ICU diaries kept by both the family and the staff.

**Methods:**

We conducted a qualitative study involving 32 semi-structured in-depth interviews of relatives of 26 patients (34% of all family members who visited patients) who met our ICU-diary criterion, i.e., ventilation for longer than 48 hours. Grounded theory was used to conceptualise the interview data via a three-step coding process (open coding, axial coding, and selective coding).

**Results:**

Communicative, emotional, and humanising experiences emerged from our data. First, family members used the diaries to access, understand, and assimilate the medical information written in the diaries by staff members, and then to share this information with other family members. Second, the diaries enabled family members to maintain a connection with the patient by documenting their presence and expressing their love and affection. Additionally, families confided in the diaries to maintain hope. Finally, family members felt the diaries humanized the medical staff and patient.

**Conclusions:**

Our findings indicate positive effects of diaries on family members. The diaries served as a powerful tool to deliver holistic patient- and family-centered care despite the potentially dehumanising ICU environment. The diaries made the family members aware of their valuable role in caring for the patient and enhanced their access to and comprehension of medical information. Diaries may play a major role in improving the well-being of ICU-patient families.

## Introduction

For family members, having a patient in the intensive care unit (ICU) is a highly stressful experience. The French FAMIREA group reported symptoms of anxiety and depression in 69% and 35% of family members, respectively; as well as post-traumatic stress symptoms in 33% [1]. Restricted visitation policies [2], [3], poor communication with the staff [4], poor comprehension of the medical information about the patient [4], participation in decision-making about patient care [5], lack of support from the staff, and fear for the patient contribute to cause distress in family members [6]. High levels of distress in family members have been reported not only during the ICU stay, but also during the next several months [7].

Strategies designed to help family members actively provide support to themselves and to the patient include broad visitation policies [8], [9], [10], involvement in nursing care [11], and participation in keeping a patient diary during the ICU stay [12], [13]. ICU diaries were introduced in the 1980s in Denmark by nurses, as a patient debriefing tool for use after ICU discharge. ICU staff participation seemed important to the effect of diaries in decreasing anxiety among family members [14]. ICU diaries are usually kept by both staff members and family members. Studies have assessed the impact of ICU diaries on long-term outcomes of patients [15], [16], [17], [18], [19], [20] and families [13]. The use of ICU diaries was associated with decreased symptoms of anxiety, depression [18], and posttraumatic stress in both patients [17] and families [12], [21] at various time points after ICU discharge. The diaries enabled survivors to make sense of their ICU experience [22]. Qualitative studies established that ICU diaries were useful in describing the patient's environment in the ICU; building a time line of health-related events; inserting the time line of the patient's experience within that of the family, community, and world events; demonstrating the continuity of the patient's life; expressing feelings and emotions; and documenting the presence, commitment, and supportive role of the staff and family [12], [23], [24]. Despite this growing empirical evidence, family members' experience with ICU diaries has rarely been investigated. To learn from family members how writing in and reading ICU diaries affected their experience in the ICU, we conducted a qualitative study based on an inductive grounded theory approach. Our findings provide new insights into the benefits of ICU diaries for families.

## Methods

### Design

We conducted a qualitative study to obtain new insights into a topic about which little published information was available. More specifically, to shed light on the families' account of their situation, we chose the grounded theory approach. The methods and procedures of grounded theory involve cycles of data collection and analysis to identify interrelations among concepts, which eventually aggregate into an integrative theory [25], [26]. The study was approved by the appropriate ethics committee (*Pitié-Salpêtrière Comité de Protection des Personnes*).

### Context

The substantive area in which we have performed our study was the 10-bed medical-surgical unit of the Saint Joseph Hospital Network in Paris, France. This ICU has placed strong emphasis on family-centred care, with around-the-clock visitation [7] and participation of family members in patient care [9]. All the patients were admitted to the same ICU and received care from the same staff.

### Content and use of the diaries

Since 2009, ICU diaries are kept by the ICU staff (physicians, nurses, and nursing assistants) and families [10]. The diaries were not part of the official clinical records of the patients. The ICU diary is in a folder placed in plain view in the patient's room. ICU staff attended lectures and discussions on writing in the diaries. There was a single diary per patient, which was used by both the staff and the family. Patients ventilated for more than 48 hours had a diary created as soon as possible by their ICU physician. All physicians in the unit could create diaries. The physician started the diary with an account of the initial medical event. Then, all staff members could write about events experienced by the patient in the ICU; they wrote in the diaries during their normal working hours, including those spent in ICU meetings about patient care. The ICU staff addressed personally to the patient. Both positive and negative changes could be recorded in the diary, although physicians were not obligated to provide complete medical information. Nosocomial and iatrogenic events were entered as part of the medical information about the patient. Relatives were invited directly to write freely in the diaries, without guidance from the ICU staff. The only instruction given to families and staff members about diary entries was to refrain from writing about confidential matters that should not be shared among the patient, all relatives, and the staff. Staff members were free to express compassion and their hope, or the absence thereof, that the patient would recover. Our previous study about the experience of the staff with ICU diaries showed that most of the staff members read the entries by the families [27]. No patients wrote in the diaries. At ICU discharge, the diary was given to the patient if he or she had recovered and to the family otherwise.

### Data collection and analysis procedures

In keeping with our focus on the family members' experience with the ICU diaries, we conducted semi-structured interviews of families' members. We used the grounded theory approach [25], [26], which involves moving back and forth between data collection and analysis until the emergence of a theory that fits the data.

The participants were selected via theoretical sampling: that is, we included individuals likely to provide the information we needed. The data obtained from the first study participants determined which new categories of participants we needed to include [28]. Thus the sample size is governed by the spectrum of data needed to develop a theory, which cannot be determined before data collection starts. The goal is not to obtain a statistically representative sample. Family members were defined as all relatives and friends who visited the patient. We interviewed families if French was their native language and their patient stayed longer than 48 hours in the ICU, and had an ICU diary. Several family members could be included for the same patient, as experience might differ according to the relationship with the patient. For each family member, we recorded age, gender, relationship with the patient, and educational level. The data recorded for each patient were age, gender, reason for ICU admission, severity of illness assessed using the Simplified Acute Physiological Score (SAPS II) based on the worst physical and laboratory data during the first 24 hours in the ICU [29] and type of admission (medical, scheduled surgical, or emergent surgical).

The main investigator (MGO) invited the family members to participate in the study. Each family member was interviewed at patient ICU discharge to ensure that the full ICU experience was captured, while avoiding the inconvenience and possible distress associated with having to return to the ICU after discharge. Prior to the interview, each participant gave written informed consent to the audio-recorded interview. An in-depth semi-directive interview was conducted by either a psychologist (MT) or a physician (MGO), both of whom were trained in qualitative data collection. The psychologist was hired specifically for the study and did not belong to the ICU staff. The physician (MGO) is on the ICU staff and dedicates most of her time to research; she does not participate in bedside patient care. In accordance with Charmaz guidelines [25], the interview guide was composed of informational questions followed by reflective and feeling-oriented questions (Table 1). Each interview lasted about 1 hour and was conducted in a quiet room in the hospital. The interviews were recorded and transcribed. To simplify the data analysis process, the data rendered anonymous only after data analysis was complete.

**Table 1:** Interview guide.

1. Can you tell me how you used the diary during your family member's stay in the ICU?
2. Can you tell me what you wrote? And perhaps also why?
3. How did it make you feel to write in the diary?
4. Do you see a connection between how the diary was used and the course of your family member's health condition?
5 Have you thought about the possibility that others will read what you wrote? How does that affect you?
6. Have you read what the staff members wrote during the ICU stay? How did that make you feel?
7. Have you talked about the diary to family members or friends?
8*. What do you think about the design of the diary (format, materials used, appearance)?

* This question was included in the first interview guide then removed after the preliminary analysis.

### First round of data collection

We first interviewed 22 family members between November 2012 and June 2013. We analysed their data and developed a provisional theory. As the study was exploratory, we used a three-step analysis process to move from a descriptive coding scheme to a more analytical one, through progressively increasing abstraction. For data analysis, we used the insider-outsider approach: researchers with experience in research in the ICU worked jointly with researchers who were new to this field. This approach strengthens the trustworthiness of research results by interconnecting interpretations made by people having different levels of understanding of both empirical research and the ICU context of care. Consequently, the analysis was done by MGO and two other individuals (AP and PM), who were not on the ICU staff and who had previously been trained in grounded theory procedures.

Open coding was the first step. We developed a fine-grained coding scheme by collaboratively analysing the interview transcripts during a workshop. Each workshop participant (MGO, AP, PM) then coded the interviews independently. The results were compared and discrepancies resolved by consensus. The first version of the coding scheme had 46 categories and sub-categories. In accordance with Creswell [30], at this stage of the analysis, we began to identify the most illustrative excerpts for later access and possible inclusion in the study report.

### Results of the preliminary analysis

Our preliminary analysis established that most interviewees both read and wrote in the diaries. Therefore, the analysis could not use one or the other of these two activities (reading and writing) as the central focus. Furthermore, the lead researcher initially felt that family perceptions about the design of the diary (format, material, and appearance) would be of interest. However, the interviewees consistently felt satisfied with the design of the diaries. Consequently, the question in the interview guide about diary design was removed. No other questions in the guide were removed, added, or changed.

### Second round of data collection

We interviewed 10 additional family members between July 2013 and October 2013. The data collection procedure was the same as for the first round, except for the removal of the interview-guide question on diary design.

We performed axial coding to view the data from a higher degree of abstraction and to uncover deeper patterns. Several subcategories were subsequently relabeled and reorganized as first-order properties of the emerging data structure. Comparison of the data from each new second-round interviewee to the existing data identified three categories, as detailed in the results section. During this phase, we also used in vivo coding (i.e., we used interviewees' words to label categories) to conceptualize the ICU experience based on the family members' perceptions. Saturation was defined as failure of data from new interviewees to generate new properties or induce changes in the data structure.

Finally, the third step consisted in selective coding to integrate and synthesise the categories derived from the analysis. In line with our research question, we paid special attention to the outcomes of the previously defined categories. These findings are reported in the results section (“Family Experience in Context” subheading). The analysis was performed in French. All quotes were translated for publication by an American physician and medical writer.

## Results

We interviewed 32 family members. Our sample was composed of 34% of the family members who visited patients ventilated for more than 48 hours. Their main characteristics and those of the corresponding patients are shown in Tables 2 and 3, respectively. The final data structure contained 13 first-order concepts, six second-order themes, and three aggregate dimensions: communication, emotional experience and humanization experience of the staff and patient (Table 4).

**Table 2:** Characteristics of the 32 family members interviewed for the study.

Variables	Data
Age in years, mean±SD	54.6±13.0
Female gender, n (%)	27 (84)
Relationship with the patient, n (%)	
Spouse	11 (34.3)
Grown child	10 (31.2)
Sibling	4 (12.5)
Other	7 (21.8)
Occupation, n (%) *	
Tertiary-sector employees**	15 (65.2)
Retired	7 (30.4)
Educational level, n (%)	
No formal education	7 (21.8)
Completed secondary education	5 (15.6)
2 years of higher education	1 (3.1)
3 years of higher education	16 (50)
5–8 years of higher education	3 (9.3)

*10 family members had never had paid jobs.

**Tertiary-sector: production of services (trade, administration, transportation, finance, services to corporations and individuals, healthcare, education, and social work).

**Table 3:** Characteristics of the 26 patients whose family members were interviewed for the study.

Variables	
Age in years, mean±SD, Median (25^th^–75^th^)	71±11, 73 [64–78]
Male gender, n%	19 (73.1)
Type of patient, n (%)	
Medical	16 (61.5)
Unscheduled surgical	8 (30.8)
cheduled surgical	2 (7.7)
SAPS II, mean±SD	49±19
Predicted hospital mortality (%)	11 (43%)
ICU stay in days, Median (25^th^–75^th^)	29 [15–44]
ICU mortality, n (%)	8 (30.8)
Reasons for admission, n (%)	
Shock	10 (38.5)
Acute respiratory disease	6 (23.1)
OPD	1 (3.9)
oma	5 (19.2)
cute renal disease	3 (11.5)
onitoring	1 (3.9)

SAPS II, Simplified Acute Physiology Score, version II; ICU, intensive care unit; COPD, chronic obstructive pulmonary disease.

**Table 4:** Data structure.

1^st^ Order Concepts	2^nd^ Order Themes	Aggregate Dimensions
Written information is more reliable than information delivered orally by the physicians.	To access and assimilate the information	Communicative experience
The use of everyday terms in the diary entries and the ability to read the entries as many times as desired improves assimilation of the information		
Cooperation between the family and staff to write the patient's story	Sharing of information	
Concern about being intrusive may be a barrier to writing in the diary		
The diary serves as a communication tool among family members		
Keeping the patient alive by expressing active feelings for him/her in writing	To bear witness to one's presence	Emotional experience
The diary allows the family members to record their presence at the patient's side.		
The diary is used as a journal in which fears and anxieties can be released	To confide and keep hope alive	
Supportive statements by the staff allow the family to project into the future, after the ICU stay.		
The diary serves as a medium for developing a warm relationship with the staff.	New way of perceiving the staff	Humanisation experience
The diary bears witness to the commitment of the staff, which goes beyond medical care		
The diary shows how the staffs take the patient into consideration.	New way of perceiving the patient	
The patient recovers the status of a living individual.		

### A communicative experience

The diaries, historically conceived as a patient debriefing tool, had major effects on family members by enhancing access to and assimilation of medical information about the patient (13/32 families members, 41%) and by helping to share various types of information among family members (5/32, 16%).

### Access to and assimilation of medical information about the patient

Although the families attached considerable importance to information delivered orally by the staff, they felt that information written in the diaries was more reliable and more powerful, particularly in the area of medical information. Family members who read entries signed by the physicians no longer felt the need to check by asking questions of other staff members. *‘There is no need to ask the same thing over and over again …we don't ask about the information in the diary, because it's written down’* (31-year-old daughter). Family members sometimes felt the information was insufficient or irregularly provided. *‘I'd like the doctor to write in the diary more often; an entry every day would work for me: I'd know what he thinks from day to day about the changes in my father's condition’* (48-year-old daughter). The family members did not focus only on the information written by the physicians: entries by other staff members were also read with interest, as they bore witness to what happened at times when no family members were in the room. *‘The diary let me know what happened at night, when I wasn't there’* (45-year-old mother). Some family members, however, seemed reluctant to read entries intended for the patient and to intrude into the intimate patient-staff member relationship (4/32, 12%) *‘When I read the entries by the staff members, I feel they're not intended for us and that I'm reading something that's none of my business’* (74-year-old spouse).

In addition to facilitating access to medical information, the families felt that the diary improved comprehension of the information, because the staff, particularly the physicians, made greater use of everyday terms for the written entries than for the oral information. Furthermore, a very powerful characteristic of the diaries was the possibility for the family members to read the entries as often as they wished, which helped them to assimilate the information. In addition, they could read the entries again at the time of their choosing and at their own pace, when the conditions were optimal for them, and they could rebuild the patient's story and assess the changes over time whenever they felt the need and regardless of the course of the patient's clinical condition. *‘Since it's written down, we can read it as often as we want to – and to be honest, I've read the diary at least 15 times, and when I read your first entry I understood it but I read it again and again because each time I needed to read everything from the beginning’* (21-year-old daughter).

### Sharing information among families members

The diary served as a channel of communication among the family members. It allowed all family members to have access to the medical information and to obtain news about the patient on a daily basis. *‘I just write that in the diary, so that the people who come to visit after me know, even if they don't necessarily have all the information’* (45-year-old sister-in-law). The main function of the diary was to provide opportunities for cooperation between the staff and family in the service of the patient and to record the medical and non-medical events that would allow the patient to re-build the gap in his or her life and the connection with the family members. *‘…so the diary lets him know what happened day by day in our family, who came to visit him, what happened to him, what progress he made’* (62-year-old spouse). Additional diary entries are provided in the online-only supplement (Table 5).

**Table 5:** Diary entries related to the communication experience.

Themes	Entries
Having access to information about the patient's health condition	Sister, 73: In general, we already knew, because they informed us, but we were glad they wrote it down and didn't hide anything, and it was in everyday language.
	Daughter, 21: Actually, in the conclusions, we had an overview of the situation, and in the entries we could discover things we hadn't heard, because it wasn't from a medical viewpoint … So it was the little details that added a bit to what I'd heard in the full meetings.
	Wife, 68: I think the diary is just perfect, because every day we can see what's new, what the doctors and nurses came to do or told him, what they think, what they wrote each time.
	Sister-in-law, 45: For me, regularly, I would read it, and that way I could get information about what was happening.
	Daughter, 21: We read everything and at least we could follow what was happening – even though we get information during the meetings and over the phone, we can see what happened, so the information is more detailed.
Assimilating the medical information	Wife, 52: We can also see the changes between the first few days when the situation was really critical, the time when we could finally really have a conversation with him when he woke up, when he recovered complete consciousness…we can see the changes, we see what's happening, we see what happened.
	Daughter, 35: So actually, I need some time to adjust. It's true that being able to settle down to write what the doctors tell us and taking the time to analyse the situation – that helps me understand the course of my father's condition … Reading makes it much easier for me to realise what's going on and makes things much more concrete, so I can understand the reality of the care that he's getting and that I don't fully grasp but that I sort of feel.
	Daughter, 51: Entries were made in it (the diary) at admission, so we knew exactly what happened when he arrived…there is no ambiguity, we don't need to ask the same questions over and over, we know what was done, what happened… so the relationship with the doctors and nurses is easier.
	Daughter, 70: I was able to grasp the medical information more serenely, because I had enough time - because I was alone, so when they talked I had to pay careful attention ….
	Sister-in-law, 62: We either softened the information given by the doctors a bit or on the contrary we catastrophized. So reading the diary corrects that and put things in their proper place.
Sharing of information	Wife, 47: People who write do so because they want to leave a message, and people who read want to receive it … I think it's really a communication tool! I'd say even a tool for communion, it brings us together…
	Sister, 51: My niece and I, the two of us come regularly, and when there's a serious difficulty, when we need to communicate – well, sometimes it serves as a connection to my niece – yes, I've left documents for her.
	Wife, 74: Writing in the diary allowed me to connect with the other members of the family.
	Husband, 68: It's interesting for the person who is in the hospital and who wants to read it to know what the experience was like.
	Sister-in-law, 62: It's really interesting to see entries by both the staff and the family members, whoever they are – I agree that it's important to have a daily record of what happened to the patient, to fill the gaps during the times he was unconscious.

### An emotional experience

The ICU stay is usually a severely traumatic experience for families. The diary helped the families understand and cope with potentially overwhelming emotional experiences. The diary produced strong emotions in the family members as they documented their presence at the patient's side, expressed their affection or love for the patient (19/32, 59%), confided their intimate feelings, and struggled to maintain hope (13/32, 41%).

### Documenting presence, expressing love or affection

The dated and signed diary entries bore witness to the presence of the family members at the patient's side. *‘That way he'll realise that he's not fighting this alone… as I explain to him in the diary, I can't be physically present – instead, I'm physically present in the diary’* (35-year-old daughter). The vulnerability of the patient encouraged the family members to say things they had never said before. *‘I felt the need to tell him that I love him, that I have always loved him, although we've been living separately for several years’* (53-year-old wife). The written diary entries served as complements to what was said to the patient. The diary created greater continuity of communication over time, by leading the family member to engage in an intimate inner dialogue with the patient. *‘Writing in the diary was life-giving for me – it's as if, since he couldn't answer me or hear me, he came to life through the writing’* (62-year-old sister-in-law). Communication usually took the form of a dialogue with the patient, although occasionally the diary served as a personal journal in which the family members confided their fears, anxieties, enthusiasm, or optimism. *‘Using the diary allowed me to resume the dialogue with him. I continued to talk to him as if he were there. And the day I started writing in the diary, I began to be able to talk to him’* (62-year-old wife). Once the dialogue was established, the personal history with the family member could continue to unfold. A connection was formed with the outside world, and the family circle concept regained its meaning. ‘*When I sit down (by his side) and write in the diary, time seems to stop and I feel I can finally settle down and take a brief look at the short day I spent with him*’ (35-year-old daughter).

### Confiding in the diary and maintaining hope

The diary offered a means of organising the inner dialogue with oneself or others, of putting names on emotions that were often powerful and overwhelming, and of expressing those emotions by incorporating them into a narrative. In this way, the writer began to find some distance from the strong emotions. *‘I also felt I was confiding in the diary. I was able to say things, my feelings for him that he couldn't hear me describe, all my pain, all the joy I felt at seeing that he was a tiny bit better…. I could open my heart to it – the diary became my confidante’* (45-year-old mother). The diary was the only document shared by the family and staff in the service of the patient. The continuous communication via the diary influenced the family's relationship with the staff, and the wishes for recovery expressed by the staff generated hope in the family members that an improvement was possible, thus changing the family's experience. *‘I believe that a doctor who writes ‘today, you were able to do this, well, I think that's far more powerful than just saying it. I believe the doctor chose his words and thought carefully about what to write, so the words really mean something So that helped me a lot’* (62-year-old wife). As shown by the verbatim passage below, another factor that maintained hope was the better emotional regulation that resulted from writing in the diary. *‘*…*It's thanks to being able to write in this diary… Writing gave me hope - it felt as if I was talking to him (in the diary), so nothing was lost.’* And the wife of this patient continued: *‘And little by little, that made me more confident, and I'd say to myself “We're going to make it, we're going to make it”’* (62-year-old wife). However, changes in the condition of the patient affected the way the families used the diary, as illustrated by the two excerpts below. In the first case, as the condition of the patient improved, his wife wrote less often in the diary. *‘It's the fact of being conscious or not – as long as he was unconscious, in an artificial coma, I used the diary to write his story … when he regained consciousness, I continued the story a bit, but with fewer details – the need wasn't as great’* (52-year-old wife). In the second case, the patient's condition deteriorated and her daughter became aware that she would be unable to read the messages. *‘It's not that I gave up, but I thought, she won't read it - she wouldn't read it, so… it's not that it was useless, not at all, but I was troubled when I took the diary this time, for the first time’* (21-year-old daughter). Additional diary entries are provided in the online-only supplement (Table 6).

**Table 6:** Diary entries related to the emotional experience.

Themes	Entries
Documenting one's presence	Wife, 62: So I'd let him know what our grandchildren were up to, that they were back in school – but I felt the most important thing was to let him know I was there.
	Wife, 54: Apart from that, the diary was a way to let him know about what happened while he was away! and to send him all the text messages he got from his friends and co-workers, to write exactly what they sent.
	Wife, 53: Words spoken are forgotten but writings remain – so I wanted him to know everything I had to say to him – that what we lived through together was very very special – I wanted him to know that, well, he was the love of my life… I wanted to confirm that I really meant what I said to him, so that he'd know, so that he could read it, so that this little diary that he will keep reminds him every day that I was there, despite all the things that happened between us, that whatever happens I'll be at his side – I'll always be at his side. So it had to be written down, so that it would stay there forever. If at any point in time I became unable to visit him here, he'd know that in spite of everything I was at his side and that I was always at his side as soon as I found out he was seriously ill – that's it.
	Daughter, 21: It was really good for me and I said to myself that if she could read it, then she'd be happy to know that we supported her and that her family held her in our thoughts.
	Daughter, 45: I write in the diary every day that I come to the ICU – I write and it's a bit like sending a message to my father at the same time.
	Wife, 72: It's as if I can be part of his life – it's life that's surrounding him, I'm here, I show him that I'm here – he senses that and maybe he'll sense it even more strongly a bit later on.
	Wife, 52: The thing is that at that time, if the person dies while in the artificial coma, we don't know what will happen – actually, we don't know any longer what kind of connection we have to the person or what will happen. And so the diary – it's really like an everyday connection, a connection through what's possible day to day – and it's the diary that allowed that connection.
	Wife, 62: Well I think it connected me to the staff – it made me feel, sort of, that I was still talking with my husband.
	Wife, 70: It's sort of also to make a connection among ourselves, the family, our children, his grandchildren. And life going on all around, life at home, cultural happenings that he's sort of putting on the back burner for now but that I try to maintain a bit.
	Wife, 52: It helped my son, my youngest who is 19 and hadn't seen his father for 15 years – and the first time he visited he didn't know what to say to him – so I said, write, then afterward you'll read it to him – so he wrote in the diary and later on he read it to his father – and I believe we say some things but when we write other things can come to mind, things we think about and that take shape as we write – but for me, I always read everything I wrote to him, I read it to him afterwards, always – he knows every word I ever wrote in the diary.
Confiding in the diary and maintaining hope	Wife, 70: A bit like a journal. To express my feelings, what I want to say to him, what I've been doing – while he's in the hospital, but me, what I do in the meantime, how I manage to get on with the difference in my life.
	Wife, 53: I need to talk, I need to speak up, and it meant a lot to me to have this diary so that I could talk about my feelings at the very moment they were there.
	Wife, 62: Actually, for me, it was more like a haven – I don't know how to explain it. To me it was almost like a private journal I could confide in - and like a hand stretched out to me – something like that, sort of like a dialogue….Because I'd read the little notes that were in the diary and when I read that today he was better than yesterday, well, that was fantastic.
	Daughter, 35: The diary meant that there was hope for later on – that's just it! (yes!) – it was exactly that connection, I'd write in it every day, because I wanted him to know what happened day by day and what he went through, him and also us, what we went through because of his treatment, and to show him what road, what journey he had travelled – to say, well, it was touch-and-go, but we can see the future, we were thinking about the future … I need to write every day, every time I visit I write to him, sometimes not much, other times I'll write two pages, sometimes ten lines or so because sometimes he's not doing very well and then it's not easy to write that he's not doing well because then we're not feeling well either – so we just write ‘you're not doing well today, and we hope tomorrow you'll be better’ – but that helps, because we hope that tomorrow will be better and that opens up the possibility of hope – it helps us move forward.
	Wife, 80: And more than anything else, it keeps our spirits up, because everyone writes a really kind note, and that feels good. It lifts our spirits, because with all the little notes by the doctors, they say ‘we took good care of you, today you're better, but this or that happened, you had a little bit of heart trouble, but it's going to sort itself out’… Every time, it boosts our spirits. They don't say ‘unfortunately, you're not doing as well as yesterday but we'll take care of you…’ It's sort of hopeful and I find that's lovely.

### A humanising experience

The last dimension of the diary-related experience as perceived by the family was humanisation of the staff, together with an awareness that the staff viewed the patient as a living human being (8/32, 25%). The diary changed the way the family members viewed the staff and, through the support expressed by the staff, changed the way they viewed the patient (8/32, 25%).

### Change in perceptions of the staff

The diary changed the families' perceptions of the staff. The contribution of the staff to keeping the diary and the time devoted by staff members to the diary outside the regular working hours were seen by the families as signs of consideration, emotional involvement, and empathy. *‘You're a doctor, you took care of her too, but you also took the time to write about your concerns and your joys and to give your encouragements, and we will never forget all that’* (21-year-old daughter). The family members no longer perceived the staff as only healthcare professionals but also saw them as people like them, since they expressed emotions. In this way, reading the diary humanised the staff members, particularly the physicians. *‘I think it's important that the doctors communicate at our level…but at least they take the time, they create a level field. It's not the doctor speaking, it's the human being, who gives information – and obviously it's medical – but it's a human being speaking to another human being’* (35-year-old daughter).

### Change in perceptions of the patients

The consideration and support given by the staff to the patient resulted in humanisation of the patient. The entries by the staff portrayed the patient as a living human being at a time when the family had difficulty recognising their loved one in the very ill and usually unconscious person connected to multiple catheters, tubes, and machines. The staff members wrote directly to the patient, describing the changes in his or her clinical condition, thus humanising the patient in the minds of the family members. *‘When I read what the staff members wrote, I really feel they're taking care of a person who is alive’* (62-year-old sister-in-law). The ICU environment and the severity of the illness cast a veil over the patient's dignity and humanity, and this veil was lifted by the writings of the staff. This change in the way the patient was perceived helped the family get through the ICU stay. ‘*The diary breathes strength into us, strength and a kind of serenity, of confidence, since what was written also was a source of confidence’* (42-year-old daughter). Additional diary entries are provided in the online-only supplement (Table 7).

**Table 7:** Diary entries related to the humanisation experience.

Themes	Entries
Change in the way staff are viewed	Wife, 70: Me, I think it helps, it really establishes a connection, a connection between you and us… it's personal and individual. It's individualised, like the care that's given here…
	Daughter, 21: I thanked one of the nurses this morning – she didn't have to write in the diary, no one had an obligation to write in the diary, but for us, for us, me personally, I was touched to see what you wrote, to see the entries… that's what we talked about with my father, that we found that really human, that for once the patient was being taken care of and the family was being taken care of too.
	Son, 41: I found the idea original and nice and that it really gave a human quality to the ICU, for the families and even more so for the patient, in our case, since my mother asked, asked insistently to see the diary … it really gives a human character to this unit, which is awfully rough.
	Daughter, 35: To see that when we aren't here, because we can't be here all the time, and fortunately they take care of my father, and I feel this emotional aspect is very important – information isn't everything – that's what I realised when a nursing aide wrote ‘I took care of you, I gave you comfort care – I did this, I gave you a massage …’, things like that! It feels good, and also they write in the diary, they take the time, the time to write, because we know that in an ICU, in intensive care, time is valuable, there's a lot to do, and I find it extraordinary that they give us some of their time.
	Daughter, 42: I don't know why, but it made me stronger – it made me stronger because I realised they were people - the people who wrote were reliable, they did something, they did what was best – and they were going to try to solve this problem.
	Wife, 75: It's a good thing that it's the doctor. The first time, I said ‘wow’ and I thought there are patients and they still take the time to write, that's it – they still take the time to write.
	Daughter, 45 (The diary is) moving, because each person leaves a bit of their heart in it and a bit of the connection they may have with him. So it's moving and it's interesting too, because the different people who write discover little things about one another. Some reveal more than others, but it's clear that for everyone some of their emotions seep into the diary.
Change in the way the patient is viewed	Daughter, 35: It helped me a lot to read what the staff members wrote, for several reasons. One was a feeling of being acknowledged, because I felt they were giving special attention to my father.
	Daughter, 42: It's that we get the feeling that we have some control over the events, it's like a log, he was given a log book so now we have a task to accomplish, finally something to do – to recapitulate what is happening – so that way we don't feel completely helpless, it helps to no longer feel so helpless when we come to see someone who – we don't know whether he can hear us, whether he can't hear us …
	Daughter, 45: Because I think we give the staff information that's rather private on the person's life … the diary also replaces those exchanges, when we can't meet. There are nurses, nursing aides, other healthcare personnel that we don't see because we don't come at the same times … but with the diary, everyone gets the information, and to take into account a person who can't communicate, who can't explain things, it recreates a connection. Let everyone take a piece of it, because everyone takes a piece, but in some way it humanises the person who is lying down in bed with all those tubes – and in a way, it reassures us, because it's also sort of for us, the fear we can have is that the kind of care that's given is a bit too… dehumanised. It's the machines that scare us – they make us realise that so much depends on the machines and on the staff, so we're not sure whether there's still a place for humanity in all that.

### The families' experience in context

**Figure 1:**
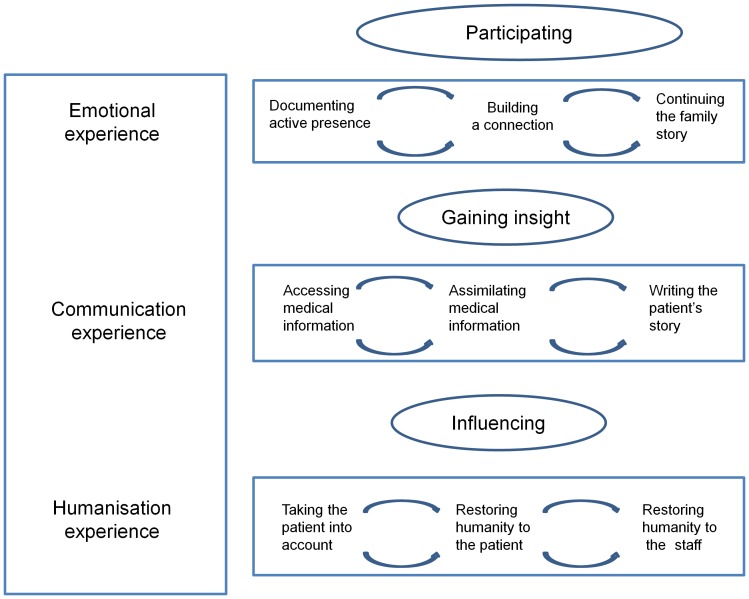
Components of the ICU-diary experience that helped to humanize the ICU experience for the families. For family members, participating in an ICU diary generated an emotional experience that built a connection with the patient and ensured continuation of the family story in the ICU. The diary enhanced the communication experience by providing medical information and describing the patient's story. The diary humanized the ICU.

In sum, grounded theory analysis of the interview data suggested the following model (Figure 1). The families perceived the diaries as promoting the acquisition and assimilation of medical information and, in collaboration with the staff, as providing an account of the patient's stay in the ICU. By documenting their presence, the diary connects the family members to the patient and ensures continuity of the family story during the ICU stay. Participating in the diary arouses strong emotions in the family members and allows them to express those emotions, thereby gaining distance and improving their emotional regulation capabilities. Finally, the diary changes the family members' perceptions of the staff and contributes to humanize their ICU experience.

## Discussion

We investigated the experience of ICU-patient family members related to ICU diaries kept by both the family and the ICU staff. We identified three main domains of the diary experience: communication, in which the diary served as an ever-present source of reliable information about the medical condition of patient; expression of emotions, which strengthened the connection to the patient during the ICU stay and helped with emotional regulation; and humanisation of both the staff and the patient.

Routinely creating ICU diaries as a clinical intervention remains controversial [19], [31]. Our study adds new insights about the benefits to families during the ICU stay and, to our knowledge, is the first investigation of this point, as most of the earlier studies focused on the patients. Analyses of the diary contents have shown how families use the diaries to communicate their emotions and feelings to the patients [12] and ICU staff [24]. Our results are consistent with this earlier work, as they indicate that reading and writing in the diaries arouse strong emotions in the family members and allow them to express those emotions. The feeling of loss due to absence of the patient from the daily family routine of communication and activities [32] was alleviated by the ability to communicate with the patient via entries in the diary. Thus, the family remained connected to the patient, who continued to be viewed as a living human being. In addition, the diary served as evidence that the family was actively present. The diary ensured that the patient remained included within the family story, so that the structure of the family remained intact. The ability to confide in the diary may have improved the regulation of emotions and contributed to the well-being of the families. This effect may explain the previously reported beneficial impact of diaries during the post-ICU period [12], [21].

Diaries improved both family-staff and within-family communication. Communication is central to family satisfaction [4]. Receiving clear medical information about the patient's status is among the most important family needs [33]. However, the high prevalence of symptoms of post-traumatic stress, anxiety, and depression, most notably within the first few days after ICU admission [7], affects the family members' perceptions of communication. Quantitative and qualitative studies established that family satisfaction was related to the organisational culture [34], quality of information [4], and perceived ICU climate. Comprehension was improved by delivery of the information in a variety of formats (oral and written) [35] and by availability of the staff for communicating with families [4]. ICU diaries provide written information in everyday language, which can be read as often as desired. Our study showed that families felt the diaries helped them to understand the patient's condition and to assimilate the medical information. ICU diary content varies across countries with or without staff participation [14], [21], [36]. In our ICU, the physicians contribute to the narration of the patient's illness in the diary. The families felt that the information written by physicians in the diaries was more powerful and more trustworthy than oral information. Thus, having the physicians contribute to the ICU diaries may help to improve the family's comprehension of the patient's situation. The diaries also improved communication within the family by ensuring that all family members had access to personal and medical information.

The families indicated that the diary was a tool of humanization of the ICU. The use of the diary by the staff to communicate with the patient indicated to the families that the staff members were compassionate and empathetic, thereby providing reassurance that the patient was safe and in good hands. Furthermore, the respect and compassion expressed by the staff generated hope in the family members. In an earlier study by our group [27] staff members reported that writing in the diaries helped them ‘to humanize their roles by emphasising the dimension of sensitivity and empathy in their interactions with the patient and the family’. In addition, awareness that the staff viewed the patient as a living human being humanised the patient in the eyes of the families. A critical illness not only causes fear that the patient might die, but also induces distress due to a perception that the patient has lost some of his or her humanity. Seen in the unfamiliar and somewhat frightening ICU environment, connected to machines, and often unable to communicate, the patient is not easily recognised by the family as their loved one, they know so well. The ability of the diary to restore the patient's humanity in the eyes of the family illustrates the differences between, and complementarity of, care and treatment. For family members who are made vulnerable by the critical illness of their relative, the diary serves as a powerful tool for improving holistic family-centred care.

Some methodological aspects are important to discuss. To take into account the possible role for subjectivity inherent in qualitative analyses, we used an insider-outsider approach, with three people, of whom two did not belong to the ICU staff and usually worked outside the ICU setting. The use of theoretical sampling with the inclusion of new interviewees in an approach that goes back and forth between the data and the analysis allows the uncovering of a theory [28]. The participation of both family members and all ICU staff members in the diaries, the specific instructions given to diary writers, and the inclusion of medical information in the diaries may limit the general applicability of our findings. The high educational level of our sample may limit the general applicability of our results. Finally, we did not take family dynamics into account.

## Conclusions

This study produced important insights about the usefulness of ICU diaries to families. The diary served as a vector that connected the patient, family, and staff into a single coherent story. It contributed to support the family members in the ICU and to restore the functional and social role of the family. The medical information given by physicians in the diary was greatly appreciated by the families, who felt it improved their comprehension. Physician participation in ICU diaries probably deserves to be encouraged.
